# Investigation of the effect of nitrate and L-arginine intake on aerobic, anaerobic performance, balance, agility, and recovery in elite taekwondo athletes

**DOI:** 10.1080/15502783.2024.2445609

**Published:** 2024-12-23

**Authors:** Zafer Kavcı, Murat Ozan, Yusuf Buzdağlı, Adem Savaş, Halil Uçar

**Affiliations:** a Atatürk University, Graduate School of Winter Sports and Sport Sciences, Erzurum, Turkey; b Atatürk University, Department of Physical Education and Sports, Kazım Karabekir Faculty of Education, Erzurum, Turkey; c Erzurum Technical University, Department of Coaching Education, Faculty of Sport Sciences, Erzurum, Turkey; d Giresun University, Department of the Food Engineering, Giresun, Turkey; e İnönü University, Department of Physical Education and Sports, Faculty of Education, Malatya, Turkey

**Keywords:** Taekwondo, lactate, nitrate, L-arginine, Wingate, lactate

## Abstract

**Background:**

Taekwondo is a complex martial art that requires speed, balance, agility, and endurance. This study aims to examine the effects of nitrate and L-arginine supplementation on acute aerobic and anaerobic performance, balance, agility, and recovery in elite taekwondo athletes.

**Method:**

This study was conducted as a double-blind, randomized, crossover study with the participation of 15 experienced taekwondo athletes aged 19.06 ± 0.96 years and 8.93 ± 1.27 years of training experience. Participants visited the laboratory a total of nine times, including a practice session and anthropometric measurements. These visits consisted of eight experimental sessions conducted at 72-hour intervals. The experimental sessions were conducted with nitrate, L-arginine, and a combination of both supplements (NIT*L-ARG) and placebo. Nitrate supplementation was provided by homogenizing fresh spinach (837.40 mg/kg), while L-ARG was given as a single dose of 6 g in powder form three hours before exercise.

**Results:**

NIT*L-ARG supplementation significantly improved the anaerobic performance of athletes in Wingate peak power and peak power (w/kg) compared to placebo and in mean power compared to NIT, L-ARG, and PLA. In addition, NIT*L-ARG supplementation significantly improved blood lactate levels and agility performance immediately after Wingate and Shuttle run tests.

**Conclusion:**

The combined intake of NIT*L-ARG was found to be effective in improving aerobic, anaerobic, and agility performances as well as fatigue levels of athletes. It was determined that taking NIT and L-ARG supplements alone contributed to the improvement of improving athletes’ performance in Wingate mean power values and subsequent fatigue level compared to PLA.

## 1. Introduction

It is widely acknowledged that sports significantly impact the human body. However, since sports consist of various disciplines, the duration, intensity, frequency, and length of ergogenic aids used according to aerobic and anaerobic energy systems form one of the most important research topics in sports sciences. Both elite and recreational athletes prefer legal methods to enhance their performance. While consuming a balanced diet is crucial for maintaining overall health and providing the necessary energy for recovery post-training, many athletes also use dietary supplements to boost performance during competitions [1].

Taekwondo, one of the most widely practiced sports in the world, requires numerous skills, high energy demands, and advanced techniques. In taekwondo competitions, athletes must possess high levels of physiological attributes [2,3]. Therefore, the rapid replenishment of anaerobic and aerobic energy stores is crucial for athletes to perform more dynamic movements, maintain high intensity during competitions, and quickly tolerate fatigue afterward [4]. Consequently, in addition to regular nutrition and rigorous training, there is a need for external nutritional aids to swiftly replenish aerobic and anaerobic energy systems, thereby reducing recovery time during or after exercise and meeting the requirement to complete above the metabolic threshold before exercise [4–6].

Today, athletes show a tendency toward natural, healthy, and functional foods. Because athletes spend significant energy in training and competitions, consuming nutritious and functional foods is essential to recover this energy. It is critical to provide energy and meet the physical and functional metabolic needs of the foods consumed. In addition, since performance-enhancing drugs are prohibited in competitive sports, performance-enhancing foods and supplements through nutrition are becoming increasingly important [7,8]. Focusing on the studies conducted in recent years, especially the effect of nitrate (NIT) and L-arginine (L-ARG) supplements on sports performance, has been a matter of curiosity. Although past studies have shown that these supplements have a positive effect on multiple parameters, there is no consensus on the results of the studies [6,9–11].

Dietary nitrate is known to dilate blood vessels and lower blood pressure by contributing to nitric oxide production. It has also been established that dietary nitrate can improve exercise performance, reduce muscle fatigue, and optimize oxygen consumption [12]. Spinach (*Spinacia oleracea L*.) has a significant nitrate content and is a popular green leafy vegetable containing many phytochemicals. Spinach is recommended as a complementary food; it contains phenolic compounds, spinacetin, patuletin, and glucuronide derivatives. It is also stated that spinach has functional properties such as antioxidant, anti-inflammatory, anti-cancer, and lipid-lowering [13,14].

Amino acids are the basic building blocks of proteins that play an important role in human nutrition. Each of them is stated to contribute differently to the structure and function of proteins. In particular, arginine, which has the highest pKa value among all amino acids and stands out with its essential and non-essential forms, is also used for different purposes [15]. L-ARG stimulates growth hormone secretion and post-exercise muscle recovery by removing ammonia from the blood during recovery from intense training [16]. These findings are supportive of research being conducted to gain a deeper understanding of the potential effects of NIT and L-ARG supplements on sports performance.

This study focused on the idea that NIT and L-ARG supplementation for the development of developing anaerobic-aerobic performance, agility, balance, and recovery performances may be effective in athletes’ high-level performance. Taekwondo athletes need to be able to sustain and maintain their anaerobic-aerobic performance, agility, balance, and recovery performances under variable competition conditions. For this reason, the aim of this study was this study aimed to determine the effects of supplemental NIT and L-ARG on the physical and physiological performances of taekwondo athletes. The main objectives of this study are to determine how the single use of NIT and L-ARG, as well as the combined intake of NIT*L-ARG and PLA, affects the performance of athletes.

## 2. Method

### 2.1. Participants

This study was carried out as a double-blind, randomized, crossover study with the participation of 15 experienced taekwondo athletes aged 19.06 ± 0.96 years and 8.93 ± 1.27 years of training experience. Inclusion criteria were (a) being over 18 years of age, (b) having at least eight years of taekwondo experience, and (c) achieving national and international rankings. Exclusion criteria: (a) use of stimulants, narcotics, and/or psychoactive substances during the test or supplementation phase, (b) consumption of substances such as nutritional supplements or steroids that may affect hormone levels in the last three months, and (c) any orthopedic, neurological, cardiovascular, pulmonary or metabolic condition that may impair performance in various tests. It was ensured that the athlete group included at least two athletes from each Olympic weight in taekwondo. Participants were informed about the research protocol, timeline, and the types of exercises and assessments they were required to complete before completing the informed consent form. All protocols and procedures were performed in accordance with by the Declaration of Helsinki.

### 2.2. Study design

**Figure 1.**
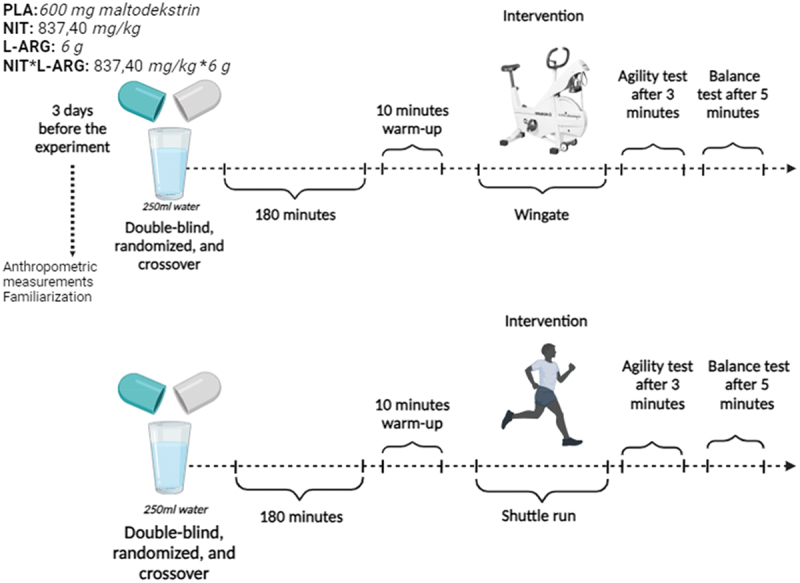
Experimental representation of the test protocol.

Participants visited the laboratory a total of nine times, including the familiarization session and anthropometric measurements. These visits included eight experimental sessions conducted 72 h apart in the same time frame (±0.5 h) to avoid bias due to circadian rhythm interference associated with supplement intake. The experimental sessions were performed in a double-blind, randomized, cross-over design with NIT, L-ARG, and a combination of both supplements NIT*L-ARG and PLA (600 mg maltodextrin). Participants were randomly assigned to a supplement group each time they visited the laboratory. They were subjected to a general warm-up for 10 minutes, followed by physical fatigue with Wingate and Shuttle run tests, agility tests three minutes later, and balance tests five minutes later (Figure 1).

### 2.3. Supplement protocol

The nitrate to be used in the study was obtained from fresh spinach. Firstly, the nitrate content of fresh spinach was determined according to Ertürk [17] and analyzed in Food Engineering laboratories. The analysis determined the amount of nitrate to be given to taekwondo athletes. The literature states that the nitrate content should not exceed 3000 mg/kg for spinach in products without heat treatment [18]. The average nitrate and nitrite values of the spinach samples used as raw materials in the current study were determined as 837.40 mg/kg and 9.20 mg/kg, respectively. According to Turkish Food Codex 2011 data, it was determined that the nitrate content of fresh spinach was below the legal limit. 50 ml of ultrapure water at 50–60°C was added to 10 g of sample, and the mixture was poured into 200 ml volumetric flasks. 50 ml of acetonitrile was added to the volumetric flask and after 15 minutes of mixing, the volume was completed to 250 ml with ultrapure water. After the prepared samples were filtered through nitrite-free filter paper, the filtrates obtained were passed through a 0.45 µm filter and transferred into vials. The amount of residual nitrate was determined using HPLC/DAD (Agilent 1100, USA). The flow rate in the column was adjusted to 2 mL/min, and measurement was performed at UV 220 nm. Hamilton PRP-X100 (5 μm × 150 × 4.6 mm, USA) was used as a column in the system, and the nitrate standard was used for identification. Results are expressed as mg/kg. Standard substances were prepared in concentrations of 1–50 mg/kg. LOD and LOQ values were obtained from the standard deviation obtained by reading the lowest concentration six times.

After the nitrate content of fresh spinach was determined for Nitrite (LOD: 1.76 mg/kg LOQ: 5.87 mg/kg) and Nitrate (LOD: 2.98 LOQ: 5.95 mg/kg), it was homogenized with the help of a blender and given to the athletes together with its pulp. On the other hand, the amount of L-arginine to be given to taekwondo athletes is given in powder form according to the usage procedure. This procedure was applied as stated at 6 g/250 ml water. Both supplements were given three hours before exercise. Until 72 hours before starting the study, participants were given a diet list that would provide a similar nutritional intake (60% carbohydrates, 30% lipids, and 10% protein). Additionally, intake of caffeine and stimulant medications was limited 48 hours before the experimental session to avoid potential interference with study results for supplement elimination. For this reason, a list was prepared for participants to stay away from NIT and L-ARG-dense foods.

### 2.4. Familiraziton

This study used the double-blinding method to prevent bias between participants and researchers. Participants were unaware of the supplements they received during each trial session ([NIT, L-ARG, NIT*L-ARG, PLA). Similarly, the researchers were not informed about which supplement was being administered. The supplement and placebo substances were prepared to be similar in appearance and taste and were administered uniformly to all participants. The spinach homogenate (NIT) and the homogenate-free placebo (maltodextrin) were prepared to resemble each other in appearance, minimizing differences in both taste and visual aspects. This ensured that participants could not guess which substance they received. The application was performed three hours before the trials, and perceptual differences due to taste and appearance were minimized during the trial process.

The nitrate was administered in the form of a specified amount of spinach homogenate, while the placebo group received 600 mg of maltodextrin. Both the nitrate homogenate and placebo were prepared to have similar volume and consistency, thus ensuring the success of the double-blinding process. Therefore, there was no perceptible difference between the homogenate and homogenate-free applications.

The success of the double-blinding process was evaluated through post-trial questions posed to the participants. Most participants could not guess which supplement they had received, demonstrating that the double-blinding method was successfully applied. The researchers were also blinded to the supplements, ensuring no bias was introduced during measurements.

The experimental protocol was carried out over eight sessions (trial sessions), with each participant randomly assigned to a supplement. The randomization process ensured that each participant was exposed to all four conditions (NIT, L-ARG, NIT*L-ARG, PLA) in equal proportions. Visits were scheduled with 72-hour rest periods and conducted simultaneously (±0.5 hours) to avoid any potential confounding effects of circadian rhythm. These measures ensured that all participants were equally represented and that the trial conditions were balanced.

### 2.5. Wingate anaerobic test

The Wingate test used a special bicycle ergometer (Monark 894E, Peak Bike, Vansbro, Sweden) designed to assess anaerobic power and capacity. During the familiarization session, participants were given detailed instructions on the correct positioning of the seat and handlebars to ensure optimal biomechanics for each individual. The seat height was adjusted to achieve a knee angle of approximately 170–175° for each participant, ensuring efficient pedaling mechanics. These personalized settings were maintained throughout all subsequent testing sessions to ensure consistency.

To further stabilize the participants’ legs, toe grips were used to keep their feet safe and stable, ensuring they remained in contact with the pedals at all times. Before the test, participants participated in a standard five-minute warm-up on the ergometer. This warm-up included pedaling at 60 watts with short five-second sprints in the second and third minutes to prepare the muscles for high-intensity effort. After the participants warmed up with a fixed warm-up protocol, they were instructed to pedal as fast as possible against a fixed resistance corresponding to 7.5% of their body weight. The test consisted of 30 seconds of full-force pedaling, where participants aimed to reach the highest possible speed and power output. The starting speed was set to zero, and participants were instructed to pedal with minimal body movement and no pre-acceleration. Throughout the test, verbal encouragement was provided by the researchers to motivate the participants to maintain maximum effort and perform at their highest during the 30-second sprint. The Wingate test collected data on peak power output and average power, which are crucial for assessing the participants’ anaerobic abilities.

Wingate was performed on a dedicated bicycle ergometer (Monark 894E, Peak Bike, Vansbro, Sweden). During the familiarization session, seat and handle positions were explained. It was modified for each participant (with a knee angle of approximately 170–175◦) and repeated in the remaining testing sessions. By using finger grippers, the participants’ legs were kept in a safe position, and they were allowed to touch the pedals. Following a five-minute warm-up at 60 W, which included five-second sprints without resistance in the second and third minutes, participants were allowed to pedal at maximum speed for 30 seconds as recommended while also exerting their maximum effort against a constant load of 7.5% [19]. Additionally, participants were instructed to crank with minimal body rotation and no pedal acceleration (initial speed was zero). Verbal incentives were used to ensure that they performed standardized tests at maximum performance.

### 2.6. Blood lactate levels

Blood samples (5 µL) were collected from the tip of the index finger of the left hand using a Lactate Scout 4 (Leipzig, Germany) analyzer following the manufacturer’s instructions. The collection process involved cleaning the fingertip with an alcohol swab to ensure a sterile environment. After allowing the skin to dry, a small puncture was made using a sterile lancet to collect the blood sample. The blood drop was then placed on the test strip of the analyzer. Lactate measurements were taken immediately after the Wingate and Shuttle run test protocols were performed to assess peak blood lactate levels resulting from physical exertion. The analyzer rapidly read the lactate concentration expressed in millimoles per liter (mmol/L). This approach provided accurate and timely measurement of lactate levels, providing insight into the athletes’ anaerobic performance and recovery dynamics.

Blood samples (5 µL) were collected from the tip of the index finger of the left hand using a Lactate Scout 4 (Leipzig, Germany) analyzer according to the manufacturer’s instructions. Lactate measurements were taken immediately after applying the Wingate and Shuttle run test protocols.

### 2.7. Balance test

The balance test was conducted using the “SPORT KAT 4000” device, and both static and dynamic balance measurements were performed. During the measurement, participants were instructed to stand on their dominant, non-dominant, and both legs and their balance performance was assessed in these positions. Static balance measurements evaluated the participant’s ability to maintain balance without movement on a stable platform, while dynamic balance assessed their ability to maintain balance on a moving platform. The balance test results were recorded using units measuring the amount of deviation on the platform (deviation values in millimeters).

Static and dynamic balance measurements were made with the “SPORT KAT 4000 Balance Measurement” device. This device can make both static and dynamic balance measurements. The platform was calibrated before each measurement. Then, the athletes were allowed to try static and dynamic balance tests so that they could familiarize themselves with the device and be more efficient in the test. In static and dynamic balance tests, dominant, non-dominant, and both leg measurements were taken.

### 2.8. Agility test

It is a test track consisting of four cones arranged in a straight line with a width of 5 m, a length of 10 m, and 3.3 m intervals in the middle. The test consists of a 40 m straight and 20 m slalom run with 180 turns between cones every 10 m. After the test track was prepared, a two-door photocell electronic chronometer system (Tumer Elektronik Ltd., Ankara, Turkey) was installed. The device has starting and ending points for measurement with an accuracy of 0.01 s. When the subjects were ready for the running position, they left the starting line of the test track, the finishing time was recorded in seconds, and the test was performed once [20].

### 2.9. Shuttle run test

MaxVO2 is a physiological measure called maximum oxygen consumption and is an indicator of aerobic endurance. The 20 m Shuttle Run test is one of the field tests used to determine this value. This test is a 23-level test in which athletes complete a 20-meter distance by going back and forth. The test starts at 8.5 km/h (9 seconds), and with each level, the running speed is increased by 0.5 km/h per minute. Athletes were asked to touch lines 20 meters apart simultaneously with the signal sound. The test was terminated if an athlete failed to reach the 20-meter range two consecutive times. Athletes continued the exercise until exhaustion. The method developed by Ramsbottom et al. was used to convert the obtained results into MaxVO2 value [21].

### 2.10. Statistical analysis

Data are presented as mean ± standard deviation. Normality distributions of the data were checked with the Shapiro-Wilk test. Analysis of variance over time (ANOVA RM) was applied for repeated measures. Greenhouse Geisser corrections for non-spherical distributions were evaluated using the Mauchly test, and pairwise comparisons between measurements were tested with Bonferroni post-hoc analysis. At the same time, the effect size was calculated with the partial eta square coefficient (ηp^2^). For all analyses, the significance level was accepted as *p* < 0.05. All statistical analyses were performed with the SPSS 21 package program (IBM Corp. Released 2012. IBM SPSS Statistics for Windows. Version 21.0. Armonk. NY: IBM Corp). Furthermore, principal component analysis (PCA) was conducted using the SIMCA 14.1 software (UMETRICS, Umea, Sweden). Principal component analysis (PCA) is a descriptive method that allows visualization of the main directions of the variability of a dataset, the relationship between samples, and between samples and variables [22,23]. In this study, Principal Component Analysis (PCA) was used to reduce the data set’s dimensionality and better understand the relationship between the basic variables. PCA aims to obtain fewer components that can explain the variance of high-dimensional data in the data set. In particular, since our data set is multivariate, PCA was preferred as a dimensionality reduction method. Before applying PCA, the data was normalized. The covariance matrix of the data was calculated, and the relationships and variances between the variables were determined. While the eigenvalues obtained from the covariance matrix represent the variance that the components can explain, the eigenvectors show the directions of these components. The components with the highest eigenvalues were selected as the components that explained the most variance. The eigenvalue (scree plot) graph was used to determine the number of components. In this graph, the remaining components were ignored from where the eigenvalues decreased rapidly. In addition, the number of components was determined using the Kaiser criterion (selecting components with eigenvalues greater than 1). The components of the first (PC1 = 67.5%) and second (PC2 = 22.9%) explain 90.4% of the total variation. These components will be used to better understand the overall structure of our model and will be associated with the main findings in the results section.

## 3. Results

This study examines the effects of PLA, NIT, L-ARG, and NIT*L-ARG supplements on the performance of elite taekwondo athletes. The research evaluates in detail the impact of these supplements on athletes’ balance, agility, and recovery performance after Wingate and shuttle run tests. The contributions of each supplement PLA, NIT, L-ARG, and NIT*L-ARG to performance parameters were compared and analyzed. In this context, the balance and agility tests of the athletes, as well as their physical recovery processes, were observed and the obtained results were evaluated. The anthropometric characteristics of the participants are given in Table 1.

**Table 1.** Characteristics of the participants.

Variables	Minimum	Maximum	Mean ± SD
Year (year)	18	21	19.06 ± 0.96
Training Experience (year)	8	12	8.93 ± 1.27
Height (cm)	160	193	179.46 ± 8.87
Body Weight (kg)	51.90	112.60	70.48 ± 15.38
BMI (kg/m^2^)	17,05	31.20	21.61 ± 3.79
Fat Mass (%)	4.01	19.00	4.95 ± 5.56
Fat Free Mass (%)	47.50	93.60	64.97 ± 11.10

**Table 2.** Lactate, agility values of participants after Wingate test in different supplement intakes.

	PLA Mean ± SD	NIT Mean ± SD	L-ARG Mean ± SD	NIT*L-ARG Mean ± SD	F	P	η_p_^2^
PP (W)	804.64 ± 201.65	823.72 ± 180.42	819.26 ± 153.25	926.25 ± 269.22^a^	4.485	.028	0.452
PP (W/kg)	11.52 ± 2.37	11.72 ± 1.40	11.71 ± 1.55	12.97 ± 1.49^a^	3.452	.010	0.521
AP (W)	504.60 ± 112.38	532.84 ± 125.63^a^	527.84 ± 118.65^a^	537.52 ± 122.85^abc^	3.756	.002	0.742
MP (W)	286.09 ± 80.33	304.40 ± 72.22	300.70 ± 66.58	318.90 ± 78.68	1.475	.632	0.124
Lactate (mmol)	11.80 ± 1.68	9.29 ± 1.32^a^	9.26 ± 2.16^a^	7.84 ± 1.69^abc^	2.586	<.001	0.801
Agility (s)	17.45 ± 1.16	17.97 ± 1.10	17.61 ± 1.18	16.76 ± 1.04^abc^	2.730	<.001	0.783

PLA: Placebo, NIT: Nitrate, L-ARG: L-arginine, PP: Peak power, AP: Average power, MP: Minimum power, ^a^: Significant difference according to PLA values, ^b^: Significant difference according to NIT values, ^c^: Significant difference according to L-ARG values.

In the repeated measures ANOVA analysis conducted with different supplement intakes, significant differences were found in the values of PP (W) (F =_4.485_, *p* = 0.028, η_p_^2^ = 0.452), PP (W/kg) (F =_3.452_, *p* = 0.010, η_p_^2^ = 0.521), AP (W) (F =_3.756_, *p* = 0.002, η_p_^2^ = 0.742), Lactate (mmol) (F =_2.586_, *p* = 0.001, η_p_^2^ = 0.801), and Agility (s) (F =_2.730_, *p* = 0.001, η_p_^2^ = 0.783). The PP (W) and PP (W/kg) values reached the highest levels after the intake of NIT*L-ARG, showing a significant difference compared to PLA values. The values of AP (W), MP (W), lactate (mmol), and agility (s) reached the highest levels after the intake of NIT*L-ARG, showing significant differences compared to the values of PLA, NIT, and L-ARG (Table 2).

**Table 3.** Participants balance scores after Wingate test in different supplement intakes.

	PLA Mean ± SD		NIT Mean ± SD	L-ARG Mean ± SD	NIT*L-ARG Mean ± SD	F	*P*	η_p_^2^
Static Double (mm)		335.80 ± 129.65	396.26 ± 247.73	382.20 ± 191.10	320.13 ± 129.63	4.231	.452	0.124
Static Right (mm)		449.26 ± 195.15	443.26 ± 194.73	436.00 ± 191.27	448.86 ± 247.65	5.412	.763	0.149
Static Left (mm)		476.80 ± 290.50	481.00 ± 271.15	487.66 ± 307.45	414.66 ± 176.74	7.421	.412	0.217
Dynamic Double (mm)		902.20 ± 114.93	947.26 ± 102.97	945.20 ± 142.53	882.33 ± 192.78	3.186	.122	0.301
Dynamic Right (mm)		1235.80 ± 242.24	1136.66 ± 346.98	1026.00 ± 228.77	901.06 ± 167.44^a^	2.693	.003	0.653
Dynamic Left (mm)		1119.00 ± 302.49	1151.06 ± 297.21	1019.93 ± 245.07	1009.06 ± 243.91	4.568	.145	0.101

PLA: Placebo, NIT: Nitrate, L-ARG: L-arginine, ^a^: Significant difference according to PLA values.

In the repeated measures ANOVA analysis conducted with different supplement intakes, significant differences were found in the values of dynamic right (F=_2.693_, *P* = 0.003, η_p_^2^ = 0.653). In the balance tests, the DN Right values reached the highest levels after the intake of NIT*L-ARG, showing a significant difference compared to PLA values (Table 3).

**Table 4.** MaxVO2 levels determined by shuttle running and lactate and agility values after shuttle running in different supplement intakes.

	PLA Mean ± SD	NIT Mean ± SD	L-ARG Mean ± SD	NIT*L-ARG Mean ± SD	F	*P*	η_p_^2^
MaxVO2 (ml/kg/min)	50.46 ± 2.92	51.89 ± 2.74	52.00 ± 2.56	54.40 ± 2.94^a^	2.163	.034	0.427
Lactate(mmol)	13.30 ± 1.90	12.82 ± 1.55	12.52 ± 1.69	8.06 ± 1.46^a^	2.756	.012	0.523
Agility (s)	17.90 ± 0.88	17.88 ± 0.93	17.74 ± 1.05	16.62 ± 0.98^a^	1.459	.029	0.471

PLA: Placebo, NIT: Nitrate, L-ARG: L-arginine, ^a^: Significant difference according to PLA values.

In the repeated measures ANOVA analysis conducted with different supplement intakes, significant differences were found in the values of MaxVO2 (ml/kg/min(F=_2.163_, *P* = 0.034, η_p_^2^ = 0.427), lactate (mmol) (F=_2.756_, *P* = 0.012, η_p_^2^ = 0.523), and agility (s) (F=_1,459_, *P* = 0.029, η_p_^2^ = 0.471). The MaxVO2, lactate, and agility values reached the highest levels after the intake of NIT*L-ARG, showing a significant difference compared to PLA values (Table 4).

**Table 5.** Balance scores after shuttle running in different supplement intakes.

	PLA Mean ± SD	NIT Mean ± SD	L-ARG Mean ± SD	NIT*L-ARG Mean ± SD	F	*P*	η_p_^2^
Static Double (mm)	370.93 ± 125.76	405.13 ± 155.48	395.33 ± 175.21	366.40 ± 112.64	4.586	.412	0.124
Static Right (mm)	487.53 ± 184.81	481.06 ± 218.41	457.26 ± 174.56	453.93 ± 232.53	5.142	.754	0.012
Static Left (mm)	523.26 ± 269.17	489.20 ± 256.28	554.33 ± 275.14	448.40 ± 163.02	3.147	.889	0.074
Dynamic Double (mm)	929.40 ± 116.95	950.20 ± 95.61	939.53 ± 142.49	894.80 ± 169.97	3.461	.456	0.132
Dynamic Right (mm)	1043.86 ± 197.48	1128.40 ± 245.74	1075.00 ± 220.87	990.00 ± 230.66	2.479	.124	0.145
Dynamic Left (mm)	1182.60 ± 256.93	1192.73 ± 216.04	1105.26 ± 203.72	1063.93 ± 196.00	4.239	.740	0.210

PLA: Placebo, NIT: Nitrate, L-ARG: L-arginine.

No significant difference was found in the balance test values applied after shuttle running as a result of ANOVA analysis in repeated measurements performed in different supplements (Table 5).

**Figure 2.**
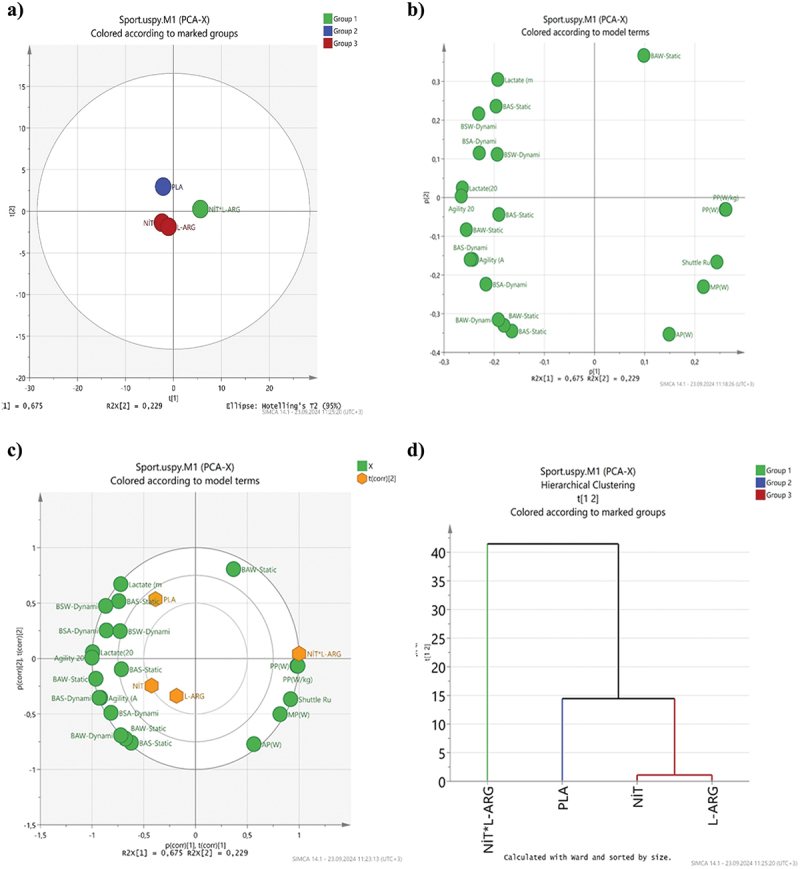
Visualizing the relationship between variables with principal component analysis (BSA: balance scores after shuttle running; BSW: balance scores after Wingate; A-Aerobic: agility after aerobic; A-Anaerobic: agility after anaerobic).

Principal component analysis (PCA) is a descriptive method that allows visualization of the main directions of the variability of a dataset, the relationship between samples, and between samples and variables [22,23]. Figure 2(a–d) shows the score scatter plot, loading scatter plot, dendrogram, and biplot principal component analysis score, and loading plots for the data of different taekwondo athletes. The first (PC1) = 67.5 and second (PC2) = 22.9 % components explain 90.4 % of the total variation. As seen in Figure 2(a–d), three different groups were formed. In addition, NIT and L-ARG were found to be in the same group, while PLA and NIT*L-ARG formed different groups. This means that NIT, L-ARG, PLA, and NIT*L-ARG contents differ from each other. When Figure 2(b) was analyzed, it was determined that the samples showed negative and positive correlations with each other. Moreover, AP (W), MP (W), MaxVO2, PP (W), and PP (W/kg) were collected on the right side, while agility, agility-20 m, lactate, and lactate-20 m were collected on the left side. Figure 2(c) shows that the highest Nitrate*L-Arginine values were found in AP(W), MP(W), MaxVO2, PP(W), and PP (W/kg) groups.

## 4. Discussion

This is the first study to investigate the effects of single and combined NIT*L-ARG intake on anaerobic Wingate performance and aerobic shuttle run performance in elite male taekwondo athletes and to evaluate balance and agility performance after fatigue. Our main findings show that the combined use of NIT*L-ARG on Wingate performance improves the performance of NIT, L-ARG, and PLA compared to PLA in PP, AP, PP (W/Kg) values, and AP levels.

Both elite and recreational athletes are interested in using legal organic and inorganic dietary supplements to enhance their performance. While consuming a balanced diet is important for maintaining overall health and fueling and recovery after training, many athletes also use dietary supplements to boost their performance during competitions [1]. The evidence base for the effectiveness of nutritional “ergogenic aids” is, as is well known, mixed, and nitrate is no exception. Beets and green leafy vegetables such as spinach, lettuce, and arugula are significant sources of naturally occurring inorganic nitrate (NO3), containing more than 250 mg of NO3 per 100 g of fresh vegetable weight [24]. Spinach, beetroot juice, and other forms of NO3 supplementation have recently become widely used nutritional interventions to improve exercise capacity and performance [25]. Among other sources rich in inorganic NO3, red spinach extract has emerged as a different alternative to beetroot juice, which contains no carbohydrates or oxalates and is rich in minerals such as potassium and magnesium [26]. Red spinach supplementation has been shown to increase plasma NO3 and NO2. Spinach extract has been shown to increase respiratory threshold in active participants, possibly indicating a greater ability to sustain aerobic metabolism as exercise intensity increases [27]. It has also been reported that red spinach extract increases the time to exhaustion during high-intensity exercise and causes an increase in exhaled NO [28]. However, recent meta-analyses support the suggestion that dietary nitrate, normally taken by athletes in the form of beetroot juice, has the potential to can potentially improve performance in various sports and exercise activities [29,30]. Previous studies reported that maximum sprint cycling performance [31,32] and 180 m sprint running performance [33] improved after NO3 supplementation, and Thompson, Vanhatalo [32] reported that NO3 supplementation could improve performance during 5, 10, and 20 m sprint runs.

The findings of our study suggest that co-ingestion of NIT*L-ARG supplementation contributes positively to Wingate performance. This is particularly important for individual and team athletes requiring high-intensity and short-duration performance. It is thought that NO3 supplementation may improve performance in exercise modes such as running and the 5–20 m sprint distances common in various team sports. This finding is important for athletes to achieve higher performance during training and competition [34,35]. Following NO3 supplementation may favorably contribute to explosive strength [36] and maximal power of the athlete’s load cycle [37]. Together with previous observations of improved sprint performance, these findings strengthen the evidence base for the use of using NO3 as a nutritional aid to enhance aspects of explosive and high-intensity performance. Improved wingate performance after NO3 supplementation may be a function of the effects of NO3 supplementation on force production: 1) in type II muscle and 2) at high contraction velocities, since maximal sprinting are velocities that require both significant type II muscle involvement and high contraction [38]. Specifically, NO3 supplementation has been shown to enhance force production or performance: 1) during the initial stages of high-frequency contractions [36]2) at high but not low contraction velocities [37,39], and 3) in type II, enhanced skeletal muscle calcium utilization in skeletal muscle in association with improved skeletal muscle calcium utilization [40]. Therefore, our results are consistent with observations of improved contractile function in studies using isolated muscle models, as noted by Thompson, Vanhatalo [32]. After dietary nitrate intake, a reduction in the oxygen cost of submaximal exercise and increased tolerance to high-intensity exercise have been consistently reported in recreationally active adults receiving both acute and chronic supplementation [41,42]. The reduction of maximal oxygen uptake without any change in exercise performance [43,44] suggests increased energy efficiency even at maximal aerobic workload. Studies in rodents have suggested that 5 to 7 days of nitrate supplementation can improve blood flow and contractile function in predominantly fast-twitch type II muscle fibers [40,45]. Our study determined that NIT*L-ARG supplementation improved the performance of athletes compared to PLA in PP (w), PP (w/kg) values and NIT, L-ARG supplementation, and PLA in average power level. In addition, it was determined that L-ARG supplementation improved the performance of athletes compared to placebo. The present study shows that PLA outputs have the lowest averages compared to the results of other nutritional supplements. Linoby, Nurthaqif [28] reported that spinach extract increased the time to exhaustion during high-intensity exercise, caused an increase in exhaled NO, and positively affected the outcomes of high-interval exercise. Although still not fully elucidated, the mechanisms purportedly responsible for the performance enhancement of athletes by nitrate supplementation may include increased skeletal muscle blood flow [45], greater metabolic recovery [46,47], and increased intramuscular calcium release [48], which may act independently or synergistically. Sandbakk, Sandbakk [33] stated that the combined intake of NIT*L-ARG supplementation had a partial positive effect on the performance of athletes compared to NIT and PLA, but the reason for these results is not fully understood. In addition, it was reported that nitrate supplementation did not improve endurance and post-fatigue lactate values in elite skiers. While the mechanisms responsible for the observed physiological effects remain unclear, the effects on muscle function and performance appear to be strong when an appropriate supplementation modality is followed [5]. In sportive performance studies in which NIT supplementation is applied, it is seen that the performance outcomes of elite athletes, intermediate athletes, and healthy sedentary people differ and are controversial even in similar studies [49–52]. Both the The training model, the dose of NIT supplementation, and the participants’ characteristics suggest that it affects performance outcomes. Although aerobic performance studies in NIT supplementation have found more place in the literature, it is also seen that field studies in terms of anaerobic performance are limited, and more studies are needed. Therefore, the results of our study are considered to be valuable.

Raymond, Yount [53] could not conclude that red spinach nitrate supplementation improved Wingate performance but found that the nitrate-supplemented athlete group had significant improvements in significantly improved lactate levels compared to the placebo group. Among the most important results of the study was the detection of a positive improvement in both anaerobic performance and blood lactate levels of NIT*L-ARG supplementation after the Wingate test compared to the results of NIT, L-ARG, and PLA. Especially in taekwondo and other combat sports (boxing, wrestling, kickboxing, karate, etc.), where anaerobic performance is used intensively, tolerating fatigue or keeping it at a low level is one of the most important markers of athletes’ success. Therefore, the role of NIT*L-ARG supplementation in improving blood lactate levels after the Wingate test is valuable for both the study and the athletes.

This study observed that the combined intake of NITL-ARG significantly improved athletes’ performance and tolerated fatigue compared to NIT, L-ARG, and PLA in terms of regarding both agility and lactate levels after the Wingate test. The dominance of the anaerobic system in the energy systems used by taekwondo athletes was crucial in the selection of selecting taekwondo athletes as the population for this study. Maintaining the decline in physical parameters after fatigue induced by anaerobic processes is essential for success in all sports, especially in taekwondo, which requires planning, quick decision-making, and more. As a result, the use of dietary supplements that directly affect these processes forms the unique nature of this study. Another significant finding of this study is that the NITL-ARG supplement improved athlete performance in the shuttle run test compared to PLA. Although there was no significant difference, the higher average VO2max results for NIT and L-ARG supplements alone compared to PLA suggest that these supplements may lead to positive developments in athletes. Additionally, it was concluded that NIT*L-ARG supplementation improved athlete performance in terms of blood lactate and agility levels after fatigue compared to the placebo.

According to the repeated measures ANOVA analysis, it was found that different supplement intakes caused significant differences in dynamic right values. This finding clearly shows the effects of supplements on muscle strength and balance performance. The fact that the NIT*L-ARG combination reached the highest levels, especially in dynamic right values , and created a significant difference compared to the PLA group can be attributed to its effect of increasing nitric oxide production. L-ARG is a precursor that plays a role in NO production and, therefore, can help carry more oxygen and nutrients to the muscles together with vasodilation. This mechanism may explain the increased balance performance [54–56].

It was observed that the NIT*L-ARG group exhibited the highest performance in agility tests. Previous studies have also reported that nitric oxide-enhancing supplements improve muscle performance. The findings of our study support these results and show that NIT*L-ARG intake increases agility values and creates a significant difference compared to PLA. This finding emphasizes the potential benefits of using supplements containing L-ARG and nitric oxide, especially to improve sports performance. Similarly, the literature shows NIT and L-ARG supplements have performance-enhancing effects [57–59]. In addition, a high effect size indicates that the effects of these supplements on muscle performance, balance, and agility are quite strong. This suggests that the combined intake of such supplements may be an effective option for athletes or individuals who want to improve their physical performance. However, similar studies should be conducted in different populations and larger sample groups to increase the accuracy of these findings.

With the increase of NO3^−^ and NO2^−^, it has been reported that PGC1-α (peroxisome proliferator-activated receptor gamma coactivator 1-alpha) [60] and AMP-activated protein kinase (AMPK) [61] are activated in skeletal muscle. These proteins are known to activate signaling pathways that support adaptive skeletal muscle remodeling in response to exercise training [62–64]. It is possible that NO-mediated signaling pathways produced by NO3- supplementation are synergistic with molecular signaling processes induced by exercise training. By enhancing transcription pathways that are integral to skeletal muscle remodeling and maintaining higher training intensities through the aforementioned effects on muscle contraction efficiency [40,65,66], dietary NO3^−^ supplementation may modulate certain physiological and exercise performance adaptations to exercise training. These processes improve athletes’ anaerobic and aerobic performance by increasing blood flow and muscle oxygenation through enhanced nitric oxide production. The effective functioning of NIT*L-ARG supplementation suggests that these biochemical pathways can significantly improve athletes’ performance.

Shannon, Barlow [67] reported that acute supplementation with nitrate-rich beet juice significantly improved 1500 m treadmill performance in elite long-distance runners and triathletes but did not enhance 10,000 m treadmill performance. Significant evidence from placebo-controlled double-blind studies supports the use of dietary NO3− supplementation to improve aspects of performance in endurance activities (e.g. cycling, running, and rowing) [68,69]. NO, a potent vasodilator, increases blood flow (i.e. tissue perfusion), which supports oxygen delivery to metabolically active skeletal muscles [50]. Additionally, it has been suggested that NO enhances skeletal muscle contractile function, indicating that more muscle work can be done per unit of time at the same metabolic cost [5,43]. Reductions in metabolic cost [6,70,71], and perceived effort during sustained submaximal and maximal aerobic exercise have likely occurred due to improvements in tissue oxygenation and contractile function resulting from dietary NO3 supplementation [72]. In light of this information, it was determined that NIT*L-ARG supplementation also played a beneficial role in enhancing aerobic performance in athletes in our current study.

The study found that the combined intake of NIT*L-ARG significantly improved athlete’s agility and lactate levels performance compared to NIT, L-ARG, and PLA after the Wingate and shuttle run tests. Rogers, Davis [58] reported that NIT supplementation significantly increased plasma NO3/NO2 compared to PLA, and in parallel, an improvement in athletes’ reactive agility performance was observed. Considering this, the improvement in agility levels among the athletes participating in our study suggests that this is a significant development for sports disciplines that rely on reactive ability for successful performance and for athletes who need to advance their reactive skills. These results are consistent with studies based on NIT supplementation, which, while not identical, are based on similar measurements [9,73,74]. The combined intake of NIT*L-ARG supplements enhances this study’s originality while limiting the ability to make concrete comparisons and explain the mechanisms of action. Therefore, more similar studies are needed in this regard. In this study, despite the lowest values being obtained in static and dynamic balance levels with NIT*L-ARG supplementation in response to fatigue induced by Wingate and shuttle run tests, the dynamic right foot balance test showed a significant improvement with NIT*L-ARG supplementation compared to the placebo. However, it was concluded that there were no significant improvements in all other parameters among the groups.

## 5. Conclusion

The combined intake of NIT*L-ARG supplementation improved the anaerobic and aerobic performance, blood lactate levels, and agility values of athletes compared to NIT, L-ARG, and PLA. However, although there were no significant improvements, supplementation of NIT and L-ARG alone contributed positively to the performance of athletes compared to placebo. Therefore, coaches and athletes may use NIT*L-ARG supplementation together to optimize performance and training. However, further research with larger sample sizes and more mechanistic study designs is required to must confirm the current findings.

## References

[1] Maughan RJ, Greenhaff PL, Hespel P. Dietary supplements for athletes: emerging trends and recurring themes. Food, Nutr Sports Perform III. 2013;29(sup1):57–20. doi: 10.1080/02640414.2011.587446 22150428

[2] Heller J, Peric T, Dlouha R, et al. Physiological profiles of male and female taekwon-do (ITF) black belts. J Sports Sci. 1998;16(3):243–249. doi: 10.1080/026404198366768 9596358

[3] Marković G, Mišigoj-Duraković M, Trninić S. Fitness profile of elite Croatian female taekwondo athletes. Collegium Antropologicum. 2005;29(1):93–99. 16117305

[4] Janowski M, Zieliński J, Ciekot-Sołtysiak M, et al. The effect of sports rules amendments on exercise intensity during taekwondo-specific workouts. Int J Environ Res. 2020;17(18):6779. doi: 10.3390/ijerph17186779 PMC7559273 32957546

[5] Jones AM, Thompson C, Wylie LJ, et al. Dietary nitrate and physical performance. Annu Rev Nutr. 2018;38(1):303–328. doi: 10.1146/annurev-nutr-082117-051622 30130468

[6] Piknova B, Schechter AN, Park JW, et al. Skeletal muscle nitrate as a regulator of systemic nitric oxide homeostasis. Exercise Sport Sci Rev. 2022;50(1):2–13. doi: 10.1249/JES.0000000000000272 PMC8677611 34669624

[7] Ozan M, Buzdagli Y, Eyipinar CD, et al. Does single or combined caffeine and taurine supplementation improve athletic and cognitive performance without affecting fatigue level in elite boxers? A double-blind, placebo-controlled study. Nutrients. 2022;14(20):4399. doi: 10.3390/nu14204399 36297081 PMC9610400

[8] Buzdağlı Y, Eyipınar C, Öget F, et al. Taurine supplementation enhances anaerobic power in elite speed skaters: a double-blind, randomized, placebo-controlled, crossover study. Biol Sport. 2023;40(3):741–751. doi: 10.5114/biolsport.2023.119990 37398976 PMC10286601

[9] López-Samanes Á, Pérez-López A, Moreno-Pérez V, et al. Effects of beetroot juice ingestion on physical performance in highly competitive tennis players. Nutrients. 2020;12(2):584. doi: 10.3390/nu12020584 32102263 PMC7071491

[10] d’Unienville NM, Blake HT, Coates AM, et al. Effect of food sources of nitrate, polyphenols, L-arginine and L-citrulline on endurance exercise performance: a systematic review and meta-analysis of randomised controlled trials. J Int Soc Sports Nutr. 2021;18(1):1–28. doi: 10.1186/s12970-021-00472-y 34965876 PMC8715640

[11] Hiratsu A, Tataka Y, Namura S, et al. The effects of acute and chronic oral l-arginine supplementation on exercise-induced ammonia accumulation and exercise performance in healthy young men: a randomised, double-blind, cross-over, placebo-controlled trial. J Exercise Sci Fit. 2022;20(2):140–147. doi: 10.1016/j.jesf.2022.02.003 PMC8904605 35308069

[12] Lidder S, Webb AJ. Vascular effects of dietary nitrate (as found in green leafy vegetables and beetroot) via the nitrate‐nitrite‐nitric oxide pathway. Br J Clin Pharmacol. 2013;75(3):677–696. doi: 10.1111/j.1365-2125.2012.04420.x 22882425 PMC3575935

[13] Singh J, Jayaprakasha G, Patil BS. Extraction, Identification, and Potential Health Benefits of Spinach Flavonoids: A Review. In: Symp A, editor. ACS Symposium Series. 2018;1286:107–136. doi: 10.1021/bk-2018-1286.ch006.

[14] Mu Y, Feng Y, Wei L, et al. Combined effects of ultrasound and aqueous chlorine dioxide treatments on nitrate content during storage and postharvest storage quality of spinach (Spinacia oleracea L.). Food Chem. 2020;333:127500. doi: 10.1016/j.foodchem.2020.127500 32693317

[15] Gupta MN, Uversky VN. Biological importance of arginine: a comprehensive review of the roles in structure, disorder, and functionality of peptides and proteins. Int J Biol Macromol. 2023;257:128646. doi: 10.1016/j.ijbiomac.2023.128646 38061507

[16] Burke L, Castell L, Stear S, et al. A–Z of nutritional supplements: dietary supplements, sports nutrition foods and ergogenic aids for health and performance—part 10. Br J Sports Med. 2010;44(10):688–690. doi: 10.1136/bjsm.2010.075218 20587641

[17] Ertürk H. Investigation of acute effects of nitrate consumption on some physiological characteristics in elite cyclists. Atatürk University; 2020.

[18] Özdestan Ö, Üren A. Nitrate and nitrite in foods. Academic Food. 2010;8(6):35–43.

[19] Bar-Or O. The Wingate anaerobic test an update on methodology, reliability and validity. Sports Med. 1987;4(6):381–394. doi: 10.2165/00007256-198704060-00001 3324256

[20] Roozen M. Illinois agility test. NSCA’s Perform Train J. 2004;3(5):5–6.

[21] Ramsbottom R, Brewer J, Williams C. A progressive shuttle run test to estimate maximal oxygen uptake. Br J Sports Med. 1988;22(4):141–144. doi: 10.1136/bjsm.22.4.141 3228681 PMC1478728

[22] Savaş A, Oz E, Oz F. Is oven bag really advantageous in terms of heterocyclic aromatic amines and bisphenol-A? Chicken meat perspective. Food Chem. 2021;355:129646. doi: 10.1016/j.foodchem.2021.129646 33892412

[23] Oz E, Savaş A, Ekiz E, et al. Polycyclic aromatic hydrocarbon content of barbecued vegetables. Çukurova J Agric Food Sci. 2021;36(1):13–22.

[24] Hord NG, Tang Y, Bryan NS. Food sources of nitrates and nitrites: the physiologic context for potential health benefits. Am J Clin Nutr. 2009;90(1):1–10. doi: 10.3945/ajcn.2008.27131 19439460

[25] McDonagh ST, Wylie LJ, Thompson C, et al. Potential benefits of dietary nitrate ingestion in healthy and clinical populations: a brief review. Eur J Sport Sci. 2019;19(1):15–29. doi: 10.1080/17461391.2018.1445298 29529987

[26] Olivares E, Peña E. Bioconcentration of mineral elements in amaranthus dubius (wild spinach, imbuya) growing wild in crops from Miranda state, Venezuela, and used as food. Interciencia. 2009;34(9):604–611.

[27] Moore AN, Haun CT, Kephart WC, et al. Red spinach extract increases ventilatory threshold during graded exercise testing. Sports. 2017;5(4):80. doi: 10.3390/sports5040080 29910440 PMC5969023

[28] Linoby A, Nurthaqif M, Mohamed MN, et al. Nitrate-rich red spinach extract supplementation increases exhaled nitric oxide levels and enhances high-intensity exercise tolerance in humans. In: editors. International Conference on Movement, Health and Exercise. Singapore: Springer; 2019. doi: 10.1007/978-981-15-3270-2_43

[29] McMahon NF, Leveritt MD, Pavey TG. The effect of dietary nitrate supplementation on endurance exercise performance in healthy adults: a systematic review and meta-analysis. Sports Med. 2017;47(4):735–756. doi: 10.1007/s40279-016-0617-7 27600147

[30] Pawlak-Chaouch M, Boissiere J, Gamelin FX, et al. Effect of dietary nitrate supplementation on metabolic rate during rest and exercise in human: a systematic review and a meta-analysis. Nitric Oxide. 2016;53:65–76. doi: 10.1016/j.niox.2016.01.001 26772523

[31] Rimer EG, Peterson LR, Coggan AR, et al. Acute dietary nitrate supplementation increases maximal cycling power in athletes. Int J Sports Physiol Perform. 2016;11(6):715. doi: 10.1123/ijspp.2015-0533 26641379 PMC4889556

[32] Thompson C, Vanhatalo A, Jell H, et al. Dietary nitrate supplementation improves sprint and high-intensity intermittent running performance. Nitric Oxide. 2016;61:55–61. doi: 10.1016/j.niox.2016.10.006 27777094

[33] Sandbakk SB, Sandbakk Ø, Peacock O, et al. Effects of acute supplementation of L-arginine and nitrate on endurance and sprint performance in elite athletes. Nitric Oxide. 2015;48:10–15. doi: 10.1016/j.niox.2014.10.006 25445632

[34] Spencer M, Lawrence S, Rechichi C, et al. Time–motion analysis of elite field hockey, with special reference to repeated-sprint activity. J Sports Sci. 2004;22(9):843–850. doi: 10.1080/02640410410001716715 15513278

[35] Spencer M, Bishop D, Dawson B, et al. Physiological and metabolic responses of repeated-sprint activities: specific to field-based team sports. Sports Med. 2005;35(12):1025–1044. doi: 10.2165/00007256-200535120-00003 16336007

[36] Haider G, Folland JP. Nitrate supplementation enhances the contractile properties of human skeletal muscle. Med & Sci Sports & Exercise. 2014;46(12):2234–2243. doi: 10.1249/MSS.0000000000000351 24681572

[37] Coggan AR, Leibowitz JL, Kadkhodayan A, et al. Effect of acute dietary nitrate intake on maximal knee extensor speed and power in healthy men and women. Nitric Oxide. 2015;48:16–21. doi: 10.1016/j.niox.2014.08.014 25199856 PMC4362985

[38] Greenhaff P, Nevill M, Soderlund K, et al. The metabolic responses of human type I and II muscle fibres during maximal treadmill sprinting. J Physiol. 1994;478(1):149–155. doi: 10.1113/jphysiol.1994.sp020238 7965830 PMC1155653

[39] Bailey SJ, Varnham RL, DiMenna FJ, et al. Inorganic nitrate supplementation improves muscle oxygenation, O2 uptake kinetics, and exercise tolerance at high but not low pedal rates. J Appl Physiol. 2015;118(11):1396–1405. doi: 10.1152/japplphysiol.01141.2014 25858494

[40] Hernández A, Schiffer TA, Ivarsson N, et al. Dietary nitrate increases tetanic [Ca2+] i and contractile force in mouse fast-twitch muscle. J Physiol. 2012;590(15):3575–3583. doi: 10.1113/jphysiol.2012.232777 22687611 PMC3547271

[41] Larsen F, Weitzberg E, Lundberg J, et al. Effects of dietary nitrate on oxygen cost during exercise. Acta Physiologica. 2007;191(1):59–66. doi: 10.1111/j.1748-1716.2007.01713.x 17635415

[42] Vanhatalo A, Bailey SJ, Blackwell JR, et al. Acute and chronic effects of dietary nitrate supplementation on blood pressure and the physiological responses to moderate-intensity and incremental exercise. Am J Physiol-Regul, Intgr Comp Physiol. 2010;299(4):R1121–R1131. doi: 10.1152/ajpregu.00206.2010 20702806

[43] Larsen FJ, Weitzberg E, Lundberg JO, et al. Dietary nitrate reduces maximal oxygen consumption while maintaining work performance in maximal exercise. Free Radical Biol Med. 2010;48(2):342–347. doi: 10.1016/j.freeradbiomed.2009.11.006 19913611

[44] Bescós R, Rodríguez FA, Iglesias X, et al. Acute administration of inorganic nitrate reduces V˙O2peak in endurance athletes. Med Sci Sports Exercise. 2011;43(10):1979–1986. doi: 10.1249/MSS.0b013e318217d439 21407132

[45] Ferguson SK, Hirai DM, Copp SW, et al. Impact of dietary nitrate supplementation via beetroot juice on exercising muscle vascular control in rats. J Physiol. 2013;591(2):547–557. doi: 10.1113/jphysiol.2012.243121 23070702 PMC3577528

[46] Dumar AM, Huntington AF, Rogers RR, et al. Acute beetroot juice supplementation attenuates morning-associated decrements in supramaximal exercise performance in trained sprinters. Int J Environ Res Public Health. 2021;18(2):412. doi: 10.3390/ijerph18020412 33430250 PMC7825729

[47] Clifford T, Berntzen B, Davison GW, et al. Effects of beetroot juice on recovery of muscle function and performance between bouts of repeated sprint exercise. Nutr, Health Athletic Perform. 2016;8(8):506. doi: 10.3390/nu8080506 PMC4997419 27548212

[48] Bender D, Townsend JR, Vantrease WC, et al. Acute beetroot juice administration improves peak isometric force production in adolescent males. Appl Physiol Nutr Metab. 2018;43(8):816–821. doi: 10.1139/apnm-2018-0050 29527927

[49] Lansley KE, Winyard PG, Bailey SJ, et al. Acute dietary nitrate supplementation improves cycling time trial performance. Med Sci Sports Exerc. 2011;43(6):1125–1131. doi: 10.1249/MSS.0b013e31821597b4 21471821

[50] Hoon MW, Jones AM, Johnson NA, et al. The effect of variable doses of inorganic nitrate-rich beetroot juice on simulated 2000-m rowing performance in trained athletes. Int J Sports Physiol Perform. 2014;9(4):615–620. doi: 10.1123/ijspp.2013-0207 24085341

[51] Peeling P, Cox GR, Bullock N, et al. Beetroot juice improves on-water 500 m time-trial performance, and laboratory-based paddling economy in national and international-level kayak athletes. Int J Sport Nutr Exerc Metab. 2015;25(3):278–284. doi: 10.1123/ijsnem.2014-0110 25202886

[52] Porcelli S, Ramaglia M, Bellistri G, et al. Aerobic fitness affects the exercise performance responses to nitrate supplementation. Med Sci Sports Exercise. 2015;47(8):1643–1651. doi: 10.1249/MSS.0000000000000577 25412295

[53] Raymond MV, Yount TM, Rogers RR, et al. Effects of acute red spinach extract ingestion on repeated sprint performance in division I NCAA female soccer athletes. Oxygen. 2023;3(1):133–142. doi: 10.3390/oxygen3010010

[54] Arefirad T, Seif E, Sepidarkish M, et al. Effect of exercise training on nitric oxide and nitrate/nitrite (NOx) production: a systematic review and meta-analysis. Front Physiol. 2022;13:953912. doi: 10.3389/fphys.2022.953912 36267589 PMC9576949

[55] Bailey SJ, Winyard P, Vanhatalo A, et al. Dietary nitrate supplementation reduces the O2 cost of low-intensity exercise and enhances tolerance to high-intensity exercise in humans. J Appl Physiol. 2009;107(4):1144–1155. doi: 10.1152/japplphysiol.00722.2009 19661447

[56] Tang JE, Lysecki PJ, Manolakos JJ, et al. Bolus arginine supplementation affects neither muscle blood flow nor muscle protein synthesis in young men at rest or after resistance exercise. J Nutr. 2011;141(2):195–200. doi: 10.3945/jn.110.130138 21191143

[57] Dewhurst-Trigg R. The effect of dietary nitrate supplementation on agility, linear sprint and vertical jump performance. (UK): University of Exeter; 2017.

[58] Rogers RR, Davis AM, Rice AE, et al. Effects of acute beetroot juice ingestion on reactive agility performance. Oxygen. 2022;2(4):570–577. doi: 10.3390/oxygen2040037

[59] Pahlavani N, Entezari MH, Nasiri M, et al. The effect of l-arginine supplementation on body composition and performance in male athletes: a double-blinded randomized clinical trial. Eur J Clin Nutr. 2017;71(4):544–548. doi: 10.1038/ejcn.2016.266 28120856

[60] Roberts LD, Ashmore T, McNally BD, et al. Inorganic nitrate mimics exercise-stimulated muscular fiber-type switching and myokine and γ-aminobutyric acid release. Diabetes. 2017;66(3):674–688. doi: 10.2337/db16-0843 28028076

[61] Mo L, Wang Y, Geary L, et al. Nitrite activates AMP kinase to stimulate mitochondrial biogenesis independent of soluble guanylate cyclase. Free Radical Biol Med. 2012;53(7):1440–1450. doi: 10.1016/j.freeradbiomed.2012.07.080 22892143 PMC3477807

[62] Coffey VG, Hawley JA. The molecular bases of training adaptation. Sports Med. 2007;37(9):737–763. doi: 10.2165/00007256-200737090-00001 17722947

[63] Lira VA, Brown DL, Lira AK, et al. Nitric oxide and AMPK cooperatively regulate PGC‐1α in skeletal muscle cells. J Physiol. 2010;588(18):3551–3566. doi: 10.1113/jphysiol.2010.194035 20643772 PMC2988518

[64] Gibala MJ, Hawley JA. Sprinting toward fitness. Cell Metab. 2017;25(5):988–990. doi: 10.1016/j.cmet.2017.04.030 28467942

[65] Bailey SJ, Fulford J, Vanhatalo A, et al. Dietary nitrate supplementation enhances muscle contractile efficiency during knee-extensor exercise in humans. J Appl Physiol. 2010;109(1):135–148. doi: 10.1152/japplphysiol.00046.2010 20466802

[66] Whitfield J, Gamu D, Heigenhauser GJ, et al. Beetroot juice increases human muscle force without changing Ca2±handling proteins. Med Sci Sports Exercise Sport Sci Rev. 2017;49(10):2016–2024. doi: 10.1249/MSS.0000000000001321 28509762

[67] Shannon OM, Barlow MJ, Duckworth L, et al. Dietary nitrate supplementation enhances short but not longer duration running time-trial performance. Eur J Appl Physiol. 2017;117(4):775–785. doi: 10.1007/s00421-017-3580-6 28251402

[68] Cermak NM, Gibala MJ, Van Loon LJ. Nitrate supplementation’s improvement of 10-km time-trial performance in trained cyclists. Int J Sport Nutr Exercise Metab. 2012;22(1):64–71. doi: 10.1123/ijsnem.22.1.64 22248502

[69] Tan R, Wylie LJ, Thompson C, et al. Beetroot juice ingestion during prolonged moderate-intensity exercise attenuates progressive rise in O2 uptake. J Appl Physiol. 2018;124(5):1254–1263. doi: 10.1152/japplphysiol.01006.2017 29357494

[70] Carriker CR, Vaughan RA, VanDusseldorp TA, et al. Nitrate-containing beetroot juice reduces oxygen consumption during submaximal exercise in low but not high aerobically fit male runners. J Exercise Nutr Biochem. 2016;20(4):27. doi: 10.20463/jenb.2016.0029 PMC5551075 28150476

[71] Vanhatalo A, Blackwell JR, L’Heureux JE, et al. Nitrate-responsive oral microbiome modulates nitric oxide homeostasis and blood pressure in humans. Free Radical Biol Med. 2018;124:21–30. doi: 10.1016/j.freeradbiomed.2018.05.078 29807159 PMC6191927

[72] Husmann F, Bruhn S, Mittlmeier T, et al. Dietary nitrate supplementation improves exercise tolerance by reducing muscle fatigue and perceptual responses. Front Physiol. 2019;10:432050. doi: 10.3389/fphys.2019.00404 PMC6491676 31068827

[73] Williams TD, Martin MP, Mintz JA, et al. Effect of acute beetroot juice supplementation on bench press power, velocity, and repetition volume. J Strength Cond Res. 2020;34(4):924–928. doi: 10.1519/JSC.0000000000003509 31913252

[74] Karampelas D, Antonopoulos K, Michailidis Y, et al. Comparison of ergogenic effects of caffeine and nitrate supplementation on speed, power and repeated sprint performance of soccer players. Physiologia. 2021;1(1):3–11. doi: 10.3390/physiologia1010002

